# Doctors' experiences on dealing with informed consent required for lifesaving interventions for pregnant women in Somalia

**DOI:** 10.3389/fgwh.2025.1584113

**Published:** 2025-08-26

**Authors:** Ahmed Aweis, Machunde Mauma, Abdulkadir Aweis, Abdulkadir Afrah, Ibraahim Abdullahi Guled, Asli Kulane

**Affiliations:** ^1^ Department of Public Health, Capital University, Mogadishu, Somalia; ^2^ Department of Global Public Health, Karolinska Institutet, Stockholm, Sweden; ^3^ Department of Research, Bidhaamiye Centre for Social Development and Rebuilding, Mogadishu, Somalia; ^4^ Department of Education & Research, Somali Maternal and Child Health Research Institute, Mogadishu, Somalia; ^5^ Department of Pediatrics, Somalia National University, Mogadishu, Somalia

**Keywords:** consent, pregnant women, male paternal members of the family, cultural practices, cultural model

## Abstract

**Background:**

Informed consent is a crucial legal and ethical requirement in the physician-patient relationship for all aspects of care. Despite, patients have the right to make their own decision in health, women in the Middle East and Africa, including Somalia, often have limited autonomy in healthcare decisions due to patriarchal structures. In Somalia, male family members including husbands frequently hold the ultimate authority in women's healthcare choices, sometimes restricting access to lifesaving sexual and reproductive health services.

**Purpose:**

To explore doctors' experiences of delay or refusal to provide consent for lifesaving interventions for pregnant women in Somalia.

**Patients and methods:**

an exploratory, qualitative design. Purposive sampling was used to select doctors working in maternity wards in the five selected hospitals. A total of 22 medical doctors were interviewed using a semi structured interview guide, and the data were analyzed using thematic analysis.

**Results:**

An overarching theme emerged: “The disconnect between healthcare system and patriarchy system” with five sub-themes namely: (1) Consent is given only by paternal male family members (2) Paternal and male witnesses signatures required for the consent form (3) Paternal male conflicts and other reasons for delaying or refusing consent (4) Potential consequences for the doctors without the consent of paternal male (5) Changing the consent guidelines from paternal male dependency. Consent of the pregnant women is given by paternal male family members since they are responsible for her life (blood/*Diya*) according to cultural practices. The husband's consent is sufficient only in the case of post-abortion care, as this also involves the fetus. Misconceptions that cesarean sections can damage the uterus, limit future pregnancies, or impair a woman's ability to perform daily activities also contribute to delayed or refusal of consent.

**Conclusion:**

This study revealed that doctors require protection when performing their duties. All doctors who participated in the study were ready to save the lives of their patients, but were assured of their safety. Patients seem to cooperate with doctors, but the cultural practices of providing consent from male members remain a challenge to the intervention. A national health policy should be drafted and approved by the cabinet that grant women the sole right to consent to life-saving medical interventions. Additionally, community mobilization is needed to educate community leaders about the negative impact of delaying or denying women informed consent to essential healthcare due to the patriarchal norms.

## Introduction

Informed consent is a legal fiduciary requirement in the physician–patient relationship in history taking, physical examination, diagnosis, and treatment. Patients have the right to make informed decisions on their health and outcomes, which requires physicians to provide the necessary information on the potential risks and benefits of the procedures and treatments (1). To obtain informed consent, the physician takes time and uses simple language to facilitate the patient's comprehension of a treatment or procedure. Adequate time is given to the patient to understand the information and make informed choices (2). For medical intervention, informed consent is widely accepted as a voluntary, uncoerced decision made by a sufficiently competent, autonomous person because of adequate information and deliberation to accept rather than reject a proposed course of action that will affect them. Consent in this sense requires action by an autonomous agent, based on adequate information and informed consent (3).

Despite the fiduciary legal requirement for informant consent in many parts of Africa, a mix of cultural bioethics and local moral obligations in the face of communal tradition ensures a mutually acceptable informed consent process (4). “Paternalism is indeed encouraged by the patients who prefer to see the doctor as all-powerful and all-knowing, and this is reinforced by the cultural practice of customary obedience to those “above you”: either in age or social rank” (5). The patients are convinced by the local moral obligation that their superiors in charge will take care of them and take full responsibility for the decisions and signing documents, while the communal culture ensures that the appointed head of a household has the power and sole responsibility for approval and disapproval of a surgery to be carried out on a younger or female member of the family (5). A study in Kashmir (6) showed a very high awareness of informed consent, but the chosen model reflected age-old medical paternalism, which would be a barrier to acknowledging patient autonomy, the basis of modern medical ethics (7). In this communal traditional environment, a mixture of cultural bioethics and local obligations can result in delays in obtaining informed consent in a timely manner. Patients who require emergency care face delays in consenting and may experience complications or death.

The World Health Organization (WHO) reports that women die every two minutes due to pregnancy and childbirth-related complications (8). Most of these deaths (almost 70%) occurred in sub-Saharan Africa, including Somalia (8). Although there have been some remarkable improvements, the maternal mortality ratio in Somalia is still very high compared to that in other countries in sub-Saharan Africa and the rest of the world (9). Recently, 692 maternal deaths per 100,000 live births in 2018–2019 which shows a decline compared with 732 maternal deaths per 100,000 live births in 2016 and 1,600 deaths per 100,000 live births in 2004–2005 (9). This indicates that much must be done to reduce maternal mortality in Somalia. It is well known that poverty, illiteracy, gender disparities, malnutrition, cultural values, and maternal behaviors are the underlying factors of maternal morbidity and mortality in sub-Saharan Africa (10, 11). Owing to these factors, women may be prevented from seeking out, obtaining, or receiving obstetric emergencies.

Accessing lifesaving interventions for pregnant mothers, such as labor induction, cesarean section, post-abortion care, removal of retained placental tissue, and blood transfusion (12), is very important in saving the lives of women and unborn children when they are all practiced without too many barriers and delays. Some low- and middle-income countries,patriarchal system and cultural practices, could play a role in influence the acceptance of live-saving interventions. Studies indicates that women in the Middle East (13) and Africa (14) have less autonomy in healthcare decisions compared to those in high-income countries (15, 16) due to patriarchal systems. In Somalia, the decision-making process for women's healthcare is often restricted, with the ultimate authority resting with their husbands. While husbands are generally reported to be supportive caregivers during times of illness, they are often restrictive regarding some of lifesaving sexual and reproductive health which might cost the life of pregnant mothers during pregnancy and childbirth (17). Furthermore, family relationships and community interconnectedness significantly impact decision-making processes in Somalia (14, 18). Despite the lack of sufficient studies in the literature on informed consent for lifesaving interventions for pregnant women in Somalia, a critical incident occurred in 2004 involving a case of disputed informed consent, where a patient presented with a uterine rupture, massive bleeding, and shock, requiring an urgent life-saving procedure. However, the paternal family members and the husband did not provide consent for this necessary intervention. Given the life-threatening nature of the situation, the patient underwent a hysterectomy to save her life. Unfortunately, this incident led to serious repercussions, including the temporary closure of the hospital due to security concerns raised by the family members and the suspension of the attending doctor (19). Furthermore, two recent studies (18, 20) conducted in Somaliland, northern region of Somalia, showed that the family must provide informed consent whenever cesarean section (CS), assisted birth, blood transfusion, labor induction, or dilatation and curettage for the removal of retained placental tissues is recommended. Therefore, this study will explore medical doctors' experiences of delays or refusal in providing consent for lifesaving interventions for pregnant women in different regions of Somalia.

This study aimed to explore doctors' experiences of seeking informed consent to conduct lifesaving interventions (post-abortion care, cesarean section, and blood transfusion) in pregnant women in Somalia.

## Material and methods

### Research design

This study adopted an exploratory qualitative design (21) that explored the experiences and in-depth understanding of factors influencing informed consent for maternal lifesaving interventions and how they deal with challenging situations in their hospitals (22).

### Setting and recruitment

The study was conducted at five referral hospitals in Somalia, two national referral hospitals, Banadir Hospital and Daynile Hospital in the capital city Mogadishu, and three regional hospitals, Galkayo Hospital, Jowhar Hospital and Kismayo Hospital based in Galmudug, Hirshabelle and Jubbaland respectively. Purposive sampling was used to select doctors working in maternal wards in the respective hospitals, with the required knowledge, experience, availability, and willingness to participate (23). The principal investigator of the project reached the public hospital directors to provide a list of doctors for contact. Doctors were then invited to be informed of the objective of the study and their voluntary participation through WhatsApp. Those consenting to participate were asked to choose the interview date and time.

### Data collection

The study used a semi-structured interview guide to capture doctors' experiences, perspectives, and process of saving the lives of pregnant women with maternal complications. Interviews were conducted in a zoom in Somalia between December 2022 and January 2023. All interviews were tape-recorded following participants' oral consent. We conducted interviews with a total of 22 doctors, comprising 16 male and 6 female physicians, until we reached saturation and no new information was being obtained (24). The interviewed doctors had a mean age of 5.5 years and represented various specialties, including obstetric gynecologists, surgeons, and anesthesiologists. Participant characteristics such as sex, age, and length of work experience are presented in Table 1, with the exception of the hospitals and regions where they were based to ensure that anonymity was maintained.

**Table 1:** Participants characteristic.

Number	Sex^a^	Age	Experience^b^
1	F	30–35	5 yrs
2	M	40–45	10 yrs
3	F	30–35	10 yrs
4	F	25–30	2 yrs
5	M	30–35	5 yrs
6	M	30–35	7 yrs
7	F	25–30	6 yrs
8	M	30–35	2 yrs
9	F	25–30	6 yrs
10	M	30–35	7 yrs
11	M	30–35	3 yrs
12	M	30–35	3 yrs
13	M	30–35	3 yrs
14	M	25–30	2 yrs
15	M	30–35	5 yrs
16	M	30–35	3 yrs
17	F	30–35	4 yrs
18	M	30–35	7 yrs
19	M	30–35	7 yrs
20	M	25–30	4 yrs
21	M	45–50	18 yrs
22	M	30–35	3 yrs

^a^ The second column of the table describes the gender of the participants, where F is female and M is male.

^b^ The fourth column of the table shows the experience is the years (yrs) of experIence in the field of the respective practices.

### Data analysis

The data were transcribed verbatim and analyzed using Braun and Clarke's six steps of thematic analysis: (i) familiarization, (ii) generation of initial codes, (iii) search for themes, (iv) review themes, (v) defining and naming themes, and (vi) write-up of themes analyzed (25, 26).

The research team thoroughly reviewed the entire dataset including detailed note-taking to ensure familiarity and initial code generation. Following this initial step of coding process, the researches focused on creating codes that accurately reflected the specific viewpoints expressed in all interviews, considering factors such as gender and medical specialty (obstetrics/gynecology, surgery, anesthesiology). We also paid close attention to the specific local context of the regions where the hospitals were located. Any unique opinions or information provided by a single participant were coded accordingly.

In the next step of the analysis, these initial codes were then combined to develop subthemes. The relevance of these subthemes and their relationship to the overarching theme were carefully discussed and refined with the research team. To effectively illustrate each subtheme, relevant quotes were carefully extracted from the interview transcripts.

## Results

An overarching theme titled “The disconnect between healthcare system and patriarchy system” and five sub-themes were identified namely: (1) Consent is given only by paternal male family members (2) Paternal and male witnesses signatures required for the consent form (3) Paternal male conflicts and other reasons for delaying or refusing consent (4) Potential consequences for the doctors without the consent of paternal male (5) Changing the consent guidelines from paternal male dependency. This information is based on in-depth perceptions and experiences of doctors.

### Consent is given only by paternal male family members

For lifesaving interventions, particularly cesarean section, doctors can only obtain consent from the paternal male family members. The main person who provides consent is her father; when he is unavailable, this responsibility is passed on to her brother, uncle, or clan leader. They are responsible for decisions of her life (blood), when it comes to matters of life and death, according to cultural practices. as stated in the following quote.

“Permission is crucial, and you must get it from special people. In Somalia there is no question we get it from her father, brother, uncle, or her clan leader, these four are the most important”. (Interview 18, M)

Doctors reported that pregnant women's maternal family members cannot be approached to provide consent. However, mothers were found to influence fathers' decision to consent. Similarly, a pregnant woman's consent is insufficient for doctors to proceed as follows:

“You must find her father to give a consent, if her father is not alive then it is her uncle or a male member of her family lineage. No permission is taken from the women. I give an example where only women came with the patient and their permission was not valid”. (Interview 11, M)

The participants in this study reported that the husband's consent was also important to obtain and sufficient only in the case of post-abortion care, since this involves the fetus. As a father, he is responsible for giving consent to his child in the same way as the patient's consent responsibility lies with her father in line with cultural practices. According to cultural practices, a husband cannot provide consent for his wife in matters of life and death. This is because he is not considered the owner of his wife's “blood.” However, he does have the right to share and enjoy other matrimonial rights and obligations with her. Consequently, in situations involving life and death decisions, consent must also be obtained from her father, as detailed below:

“The reason why we take only husbands in this case [post-abortion] because the fetus is the husband's responsibility”. (Interview 9, F)

“The husband has the culture {matrimonial relationship and obligations} of the wife but not her blood {decisions of her life, when it comes to matters of life and death}” (interview 11, M)

“Since the blood {decisions of her life, when it comes to matters of life and death} of the wife does not belong to the husband and only her culture {matrimonial relationship and obligations} belongs to her husband. We give the decision to her family, and if the child is in trouble, it is the husband that is responsible”. (interview 3, F)

Furthermore, if paternal male family members cannot be reached or conflict with their husbands, the doctor informs hospital management. If these issues are not resolved, the criminal investigation department (CID) is contacted to decide upon and give permission. Some participants shared their experiences of dealing with this situation.

“Permission is required. It is linked to her family and husband. If they are not available, we reach out to the hospital management who then forward it to the Criminal Investigation Department (CID). CID gives written permission to operate”. (Interview 21, M)

### Paternal and male witnesses signatures required for the consent form

Consent is crucial for lifesaving interventions for pregnant women. Participants were talking about objecting to lifesaving interventions such as labor induction, post-abortion care, and blood transfusion. According to doctors, all these conditions are very important and require consent before they conduct any lifesaving intervention for pregnant women. Due to different circumstances, they are objecting to labor induction, post-abortion care, or blood transfusion. All participants agreed that doctors must obtain permission before performing any lifesaving interventions such as labor induction, post-abortion care, and blood transfusion. Even if the woman is ready and accepted, without the consent of the given members of the woman's family or husband, the doctors cannot do anything. One participant stated:

“When we are in Somalia there are challenges in surgery for mothers whether emergency or planned, you always require permission”. (Interview 20, M)

Doctors emphasize that it is crucial to document consent according to hospital guidelines. Doctors obtained written consent from three signatories (two males and the pregnant woman) and two witnesses. In cases where the responsible consent is not present in the hospital, it is documented by recording the voice, text messages, and online video as one participant illustrated it clearly.

“If her father is not in the city. We take the consent on voice recorder, but we conduct separate interviews with the patient and her father to ascertain he is the right person. We take signature and thumb print and two witnesses”. (Interview 21, M)

### Paternal male conflicts and other reasons for delaying or refusing consent

All doctors indicated that there were sometimes conflicts that stopped or delayed the lifesaving interventions. Conflicts between husband and wife (pregnant woman), husband and father-in-law, or fighting from both families may be reasons for delay or refusal of consent In cases where the husband consents to the procedure, but the wife's family refuses, the resulting conflict can tragically lead to the death of the pregnant woman as one doctor stated:

“Sometimes we see husband asking/begging to save his wife's and baby's lives and her father refusing. Father and family say we will take her, and we will read the Quran on her. The husband felt extremely helpless. Sometimes both father and husband refuse. Sometimes they waste time and bring her back in much worse situation”. (Interview 21, M)

Normally the husbands has only the marital relations, {Matrimonial relationship and obligations} with the wife while for her blood {decision of her life, when it comes to matters of life and death} it is her father that is responsible for. (interview 1 F)

On the other hand, conflicts between husband and wife, where they may be going through divorce or other issues even when her paternal male is willing, the husband can refuse to give consent because he is responsible for the fetus and that can be a threat to the pregnant woman's life, as expressed here:

… there is a dispute between her and her husband. When called, the husband told the doctor “ if you touch the baby inside her, I will take you to court”. (interview 21 M)

Delaying and/or refusing consent in emergency situations exposes patients to complications. Doctors indicated that there is a misconception about surgery, which is perceived to include death and disability of the pregnant women and money generating opportunities for hospitals and doctors. As doctors reported in this study, it is believed that surgery may damage the uterus and limit the number of babies she can have in the future, as well as weaken her strength, thus preventing her from performing her daily activities. The quotes below show:

“We encounter this refusal of the permission. They worry about her uterus not working later to have more babies or the risk of removing her uterus”. (Interview 20, M)

“Since the mother is the main active person in the house. If she goes through surgery, she may not be able again to do her usually house chores, cannot even carry three litres container”. (Interview 9, F)

Participants also mentioned that performing surgery benefits doctors and hospitals by making more money and training new young doctors. One doctor stated:

“The surgery makes money for the hospital or is for training the students. You become disabled if you are operated on several times”. (Interview 3, F)

Furthermore, most doctors noted that consent also declined for blood transfusion for several reasons, such as religion and the belief that blood from someone else can cause a pregnant woman to be unwell.

“Even blood transfusion needs permission. They fear it may cause a feeling of heat/hot. Some think religiously it is wrong to give blood. Some say we can nourish her, and her blood will improve. Feeling hot is understood that the person may not feel certain things or may get disoriented”. (Interview 17, F)

“They don't want to allow their patient to receive strangers blood either”. (Interview 20, M)

According to doctors, some parents delay giving consent by looking for other non-medical treatments such as religious and traditional practices.

“Yes, people refuse but we try to explain the risk involved if she doesn't get the surgery and the majority accept. But it is not a lot. It usually happens due to them insisting that she can deliver normally. So, explaining and convincing them takes a lot of time and then complications that could be avoided occur and mother dies”. (Interview 19, M)

Most participants stated that traditional birth attendants (TBA) were part of the problem when it came to delays. TBAs are trusted advisers who accompany women and play a crucial role in influencing their decisions. As this doctor commented:

“TBA midwives are problematic. They come accompanying the patient, they are difficult to identify. They change your advice and advise them differently”. (Interview 9, F)

### Potential consequences for the doctors without the consent of paternal male

Doctors have illustrated the consequences of lack of consent during lifesaving interventions for pregnant women. Most participants felt helpless or powerless when they did not obtain consent on time or when responsible people refused to give it. Doctors claimed that they had the desire, skills, equipment, and knowledge to help and save the lives of pregnant women and their children, but sometimes they could not because of delayed or denied consent. One participant stated:

“I felt bad hope for a patient I can serve and there is no signature. If you do have the chance to help, you do not have full power, and it is painful to come to us from time to time. And we can't do it”. (Interview 3, F)

In the case of high-risk death for a pregnant woman, even with her consent, if the doctor performs the intervention, they are likely to face possible consequences such as imprisonment, “*diya*” (blood money), or death. Doctors were very concerned about the safety and dangers of being harmed by the family members of pregnant women. Doctors were very emotional when they elaborated on the possibility of being harmed or killed while saving their lives, as stated below.

“Without permission, you can face many problems. They can hit/harm you in the hospital, even in the surgery room. They can harm you outside the hospital. Or they can take you to court. That is why we inform the hospital management”. (Interview 13, M)

Participants described that members of the family of the pregnant women may sometimes take the doctor to court if they do surgery without their consent. Even if a doctor does a surgery and it is successful, but you do not get the proper consent from the father or male member of her family lineage, they may go to court, which you can send to prison. Some doctors were imprisoned because they did not provide consent.

“In an emergency case, someone who works at the hospital who knows the family but is not related gave the permission. The surgery was successful. But family put the man in prison, even though they know him very well and both mother and child survived. Imagine what would have happened the management that could get you of the problem is not there”. (Interview 15, M)

### Changing the consent guidelines from paternal male dependency

Participants expressed ways to overcome challenges with their consent. Their suggestions range from the individual, community, institutional, and government levels. At the individual level, all doctors suggested that women be allowed to provide consent. According to doctors, in emergency situations, it is risky to find other members of the family to provide consent, while pregnant women are in a critical condition and need quick intervention. Some doctors have emphasized the following.

“Women to be empowered, once the mother [pregnant woman] is present, no signature is required”. (Interview 3, F).

“The patient can give the signature and no need for someone else”. (Interview 4, F).

Doctors have suggested that public awareness should help the community understand consent and how it works. Participants believed that most citizens were unaware of the actual situation during obstetric emergencies. Some participants suggested that people should be educated that surgery is not a business, and that doctors and hospitals do not make money out of it. Doctors also suggest that the community should be informed about the risks of refusing surgery when a pregnant woman is in a critical condition.

“Public awareness on the radio and television. Then one can explain more easily to them when the situation appears. The elders, community leaders who are the community eyes, the TBAs, teachers and religious leaders to assembled and informed about risk the mother faces. The ministry of health, the states must allow everyone is responsible for themselves before their parents and that s/he can decide for themselves. The parliament must approve it”. (Interview 13, M)

Doctors elaborated on the importance of being protected by the country's laws. Doctors want the Somali government to formulate a law to protect them when they perform surgery to save pregnant women and their unborn children. One doctor stated:

“Government should set up an office to defend us or to produce a legal process so we can save lives, allow the person suffering to give permission and it is unfortunate to give that responsibility to someone else”. (Interview 6, M).

Another doctor wanted these changes to be discussed among health experts in the whole country, and then came up with a policy supported by law.

“I would suggest that experts from the whole country gather and debate about this issue. Then come out with points to take up with the government so law is made about this. The community including elders should also be involved”. (Interview 13, M)

## Discussion

The findings of this study are summarized into one overarching theme “The disconnect between healthcare system and patriarchy system” under five themes, namely: (1). Consent is given only by paternal male family members, (2). Paternal and male witnesses signatures required for the consent form, (3). Paternal male conflicts and other reasons for delaying or refusing consent, (4). Potential consequences for the doctors without the consent of paternal male, (5). Changing the consent guidelines from paternal male dependency.

Cultural practices influence the decision of lifesaving interventions for pregnant women in Somalia.

Due to the limited reach of state authority and the reinforcing influence of state law enforcement and judicial systems, customary justice, or “*xeer*”, functions as the main way many Somalis access justice (27). This informal justice system (*xeer*) and the Somali and culture is male-centered, similar to other countries in Sub-Saharan Africa, where men make the most decisions and have greater power than women (28–30). Cultural practices are powerful and larger than those of women in these societies. “A woman belongs to her father's clan world. Even if the woman marries a man from another clan and moves to another clan's territory, the woman remains a member of her father's clan” (31). Women are supposed to follow decisions made by their husbands or male paternal family members (32). Although her male paternal family members make decisions about pregnant women, sometimes behind the scenes, wives or female members might influence their husbands to make decisions. The final decision must be made by the male members. Cultural practices influence families in making decisions that affect pregnant women's health outcomes (33). This is similar to the present study; doctors reported that pregnant women have no choice but to follow cultural practices, whereby male members give consent for lifesaving interventions.

The findings of the present study are similar to those of other studies (18, 20) carried out in Somaliland, which argue that the consent for post-abortion care and blood transfusion is given by the husband, but surgery to a pregnant woman is the responsibility of her male paternal family members. Other studies have reported that pregnant women in northern Nigeria should obtain permission from their husbands to seek care in the hospital (34). In contrast, other studies (35–38) find opposite by report that women over the age of 18 make their own decisions regarding blood transfusion and cesarean section without restrictions from their husbands or parents.

The present study found that family members delay and refuse consent for several reasons, for example, a misconception about surgery, which includes the death of the pregnant women, disability, and the generation of more money for hospitals and doctors. This finding is similar to other studies (39–41) in which the participants shared the same experiences, believing that cesarean delivery to women limits the ability of a woman to carry out household responsibilities, causes disability, and can result in death. According to a study by Brown et al. (40), women reported concerns about disability (being unable to work) after a cesarean section (CS), whereby they believed in getting a baby by natural birth, and within a few days you could start working. Women argued that those who delivered a baby by cesarean section could take more than four months to recover. Similar to the present study, previous studies in China, Chile, and Sub-Saharan Africa have described the belief of the participants about doctors and hospitals making more money to perform cesarean sections (60–66) (42–45). On the contrary, in Iran, Shirzad et al. found that pregnant women family members, husbands and women themselves prefer to undergo cesarean section and reasons given by women is that their husbands' concerns about sexual function (46).

Family members of pregnant women may refuse to give consent for blood transfusion with someone else because it can cause death to the pregnant woman, making them weak and mentally ill. This finding is similar to that of previous studies conducted in Nigeria and Saudi Arabia, which reported that participants preferred the donor to be a direct donor, either a family member or friend, for fear of acquiring blood-borne infections (47, 48). Many previous studies in other parts of the world have reported the refusal of consent for blood transfusion, but the reasons given were mainly associated with religious reasons and not medical or traditional beliefs, as shown in the present study (49, 50).

The relationships and expectations domain supports the idea that doctors are part of both the health system group and the patriarchal community system, which places them in difficult situations since the civil war erupted. The present study indicated that doctors are worried about their safety because sometimes family members of a pregnant woman attack them physically or kill them with guns if they do not obtain proper consent to perform a Cesarean Section. This finding is supported by studies conducted in Turkey (51), Bangladesh (52, 53), and Nepal (54). Abdillahi et al. reported that doctors in Somalia were threatened to be killed by their husbands when trying to perform a cesarean section without consent (18). Doctors in the present study were also worried about payment of a “*diya*” blood money as a compensation or reconciliation for the death or injury of a woman after a cesarean section without a proper consent. “*Diya*”—Blood money—is still practiced in Sudan, Afghanistan, Yemen, South Sudan, and Somalia but the purpose and process of doing it differ in each country (55). A previous study in Somalia reported that among the Issa clans of North Western Somalia, if a doctor kills a woman during a cesarean section, he/she will be charged to pay 50 camels as a “*diya*” blood money (56). “Fifteen camels are given to the family of the deceased while the rest are distributed to the clan members according to the share, they contributed during the collection of blood money” (56). Many doctors in Somalia could not afford to pay 50 camels because of their low salary.

Doctors in the present study suggested very important things to improve the situation of current consent, focusing on the women themselves, community, institutions, and government of Somalia. Consistent with the present study (18, 20), it is crucial for the government of Somalia, through the Ministry of Health, to protect doctors dealing with pregnant women in emergency lifesaving interventions by addressing the challenges of obtaining consent and ensuring theft after surgery. El-Sadig et al. (57) reported that the current government of Sudan has established a new law and police force to protect healthcare workers and punish those who abuse them through imprisonment and paying fines. This initiative has reduced the amount of violence against doctors in Sudan. Similar to findings from Ethiopia (58, 59) and Tanzania (60), the present study also shows that there is poor awareness of obstetric danger signs among pregnant women and family members, which calls for addressing this issue.

Doctors feeling powerlessness or helpless because they feel that they have knowledge, skills, and desire to help save lives of pregnant women, but they couldn't do that when they family members of pregnant women refuse to give a consent on time. This finding is in line with a study conducted in Somaliland (20), which reported that healthcare providers had the capacity and skills to perform emergency cesarean sections, but family members refused to give consent in time, which could result in maternal deaths. Furthermore, this phenomenon can be characterized as an additional delay, or a fourth delay, beyond the three delays identified in the Thaddeus framework, which contributes significantly to maternal complications and death in the country (61). The limitation of this study is that it exclusively interviewed doctors. As a result, the findings are based solely on their perspectives. To gain a more comprehensive understanding of consent, future research could benefit from including the viewpoints of other key stakeholders such as husbands and parents of pregnant women, clan leaders, government officials, and religious leaders.

## Conclusion

The research has revealed that doctors require protection when they perform their duties. All doctors who participated in the study were ready to save the lives of their patients, but were assured of their safety. Patients seem to cooperate with doctors, but the cultural practices of providing consent from male members remain a challenge to timely lifesaving interventions.

The potential risks to patient lives necessitate the intervention of the national health policy, by advocating to draft a policy and approved by the cabinet that grants women the sole right to consent to life-saving interventions that they need without requiring the consent of their male next of kin. In addition, to formulate a law to protect healthcare providers who perform emergency life-saving surgery during critical situation on pregnant women and their unborn children without the consent of family members. On the other hand, community mobilization is crucial to ensure that elders, father and husbands who adhere to statutory laws (“xeer”) understand the detrimental consequences of delays and refusals of life-saving interventions for women by men, which perpetuate a patriarchal system that restricts women's bodily autonomy.

Further research is needed to explore the views of all community stakeholders, such as the traditional elders, religious leaders, and women.

## Data Availability

The raw data supporting the conclusions of this article will be made available by the authors, without undue reservation.
